# Application of 3D tumoroid systems to define immune and cytotoxic therapeutic responses based on tumoroid and tissue slice culture molecular signatures

**DOI:** 10.18632/oncotarget.19965

**Published:** 2017-08-05

**Authors:** Niklas K. Finnberg, Prashanth Gokare, Avital Lev, Sergei I. Grivennikov, Alexander W. MacFarlane, Kerry S. Campbell, Ryan M. Winters, Karen Kaputa, Jeffrey M. Farma, Abbas El-Sayed Abbas, Luigi Grasso, Nicholas C. Nicolaides, Wafik S. El-Deiry

**Affiliations:** ^1^ Department of Hematology/Oncology and Molecular Therapeutics Program, Laboratory of Translational Oncology and Experimental Cancer Therapeutics, Fox Chase Cancer Center, Philadelphia, PA, USA; ^2^ Cancer Prevention and Control Program, Fox Chase Cancer Center, Philadelphia, PA, USA; ^3^ Division of Basic Science, Fox Chase Cancer Center, Philadelphia, PA, USA; ^4^ Biosample Repository Facility, Fox Chase Cancer Center, Philadelphia, PA, USA; ^5^ Division of General Surgery, Department of Surgical Oncology, Fox Chase Cancer Center, Philadelphia, PA, USA; ^6^ Division of Thoracic Surgery, Department of Surgical Oncology, Fox Chase Cancer Center, Philadelphia, PA, USA; ^7^ Morphotek Inc., Philadelphia, PA, USA

**Keywords:** 3D, organoid, colorectal cancer, immune cells, biomarkers

## Abstract

We have developed 3D-tumoroids and tumor slice *in vitro* culture systems from surgical tumor specimens derived from patients with colorectal cancer (CRC) or lung cancer to evaluate immune cell populations infiltrating cultured tissues. The system incorporates patient's peripherally and tumor-derived immune cells into tumoroid *in vitro* cultures to evaluate the ability of the culture to mimic an immunosuppressive tumor microenvironment (ITM). This system enables analysis of tumor response to standard therapy within weeks of surgical resection. Here we show that tumoroid cultures from a CRC patient are highly sensitive to the thymidylate synthase inhibitor 5-fluorouracil (adrucil) but less sensitive to the combination of nucleoside analog trifluridine and thymidine phosphorylase inhibitor tipiracil (Lonsurf). Moreover, re-introduction of isolated immune cells derived from surrounding and infiltrating tumor tissue as well as CD45+ tumor infiltrating hematopoietic cells displayed prolonged (>10 days) survival in co-culture. Established tumor slice cultures were found to contain both an outer epithelial and inner stromal cell compartment mimicking tumor structure *in vivo*.

Collectively, these data suggest that, 3D-tumoroid and slice culture assays may provide a feasible *in vitro* approach to assess efficacy of novel therapeutics in the context of heterogeneous tumor-associated cell types including immune and non-transformed stromal cells. In addition, delineating the impact of therapeutics on immune cells, and cell types involved in therapeutic resistance mechanisms may be possible in general or for patient-specific responses.

## INTRODUCTION

Historically, the successful establishment of exponentially growing viable cells from patient-derived tumors in *in vitro* cell cultures has been found to occur from a minority of tumor tissues following artificial selection of a sub-population of tumor cells. Such limitations make it difficult to model patient variability in drug responses *in vitro*. Moreover, the lack of stromal cells that play a critical role in tumor viability, growth and drug response *in vivo* make the prediction of tumor response of immune-based therapeutics challenging. Through recent advances in three-dimensional (3D) culture (“tumoroid”) methodologies some of these hurdles are beginning to be addressed. However, it is currently unclear to what extent such models can be engineered to retain important phenotypic properties of infiltrating immune cells from the tumor tissue of origin that mimic the *in vivo* environment.

A long-standing desire has been to test tumor tissues from patients to rapidly prognosticate treatment outcomes *in vitro* and develop treatment strategies that account for inter- and intra-patient tumor heterogeneity. Although the methodology to establish tissue *in vitro* cultures has been available for more than a century [1], the use of two dimensional (2D) culture techniques to establish continuous tumor cell lines from explanted tumor tissue cultures is hampered by artificial culture conditions that severely diminish the success rate, and tumor clonal heterogeneity. Further, time constraints render this approach impractical for clinical application to derive drug response profiles of tumors for specific patients. Recent advances in patient-derived 3D cultures have facilitated what is perhaps more relevant modeling of cancer *in vitro* and this can be combined with whole-genome sequencing approaches to couple functional and correlative tumor-specific data with the promise to shape the landscape of precision medicine. 3D *in vitro* tumoroid cultures can be rapidly established from biopsied or resected tumor tissues and subsequently subjected to *in vitro* sub-cultivation, expansion and genomic manipulation using e.g. CRISPR/Cas9 methodology [2]. Patient-specific tumoroid response profiles have been established in relation to their genomic profiles following high-throughput screening against cytotoxic drug libraries [3, 4]. Clinical trials are currently ongoing to determine whether this approach can prognosticate treatment outcome of pancreatic, liver and colorectal cancer patients [5, 6]. Interestingly, tumoroid cultures also maintain the tumor heterogeneity of the tumor-of-origin and since normal surrounding tissues can be established in addition with little modification to the culture media, a patient disease-specific genomic or proteomic tumor profile can be established in relation to the normal tissue from which the tumor arose [5, 7]. Tumoroid cultures can also be implanted orthotopically in immunodeficient mice to address the impact of disease-specific driver genes and their impact on the tumoroid's capacity to metastasize to distant organs [8].

Although tumoroid 3D cultures allow the expansion of the tumor epithelium they are typically devoid of non-epithelial stromal cells from the tumor-of-origin and therefore their use is limited in addressing therapeutic strategies targeting this compartment. The ability to retain tumor specimens with stroma is important to establish models that can represent *in vivo* tumor biology with a microenvironment including stromal and immune cells that play a prominent role in modulating processes such as tumor progression and immune evasion [9–12]. Alternative *in vitro* culturing methodologies have been employed to maintain the heterogeneous tumor microenvironment by embedding tissue fragments in a 3D culture matrix in an air-liquid interface culture system (ALI) to maintain a polarized epithelial cell layer in close proximity to stromal cells. This culture system has been shown to yield expanding cystic structures from neonatal small intestine for up to 350 days in culture [13]. The wall of the intestinal cysts consists of a polarized epithelial monolayer with an apical inner luminal surface and a basal outer surface in close proximity to alpha-smooth muscle actin expressing myofibroblasts containing fully differentiated microstructures of cultured intestinal epithelial cells when expanded in collagen [13]. Furthermore, this ALI model system may be used for oncogenic *in vitro* transformation of primary epithelial and mesenchymal explanted colon, stomach and pancreatic mouse tissues from mice [14].

Here we have optimized a novel 3D culture methodology to investigate and characterize resected tumor tissue in the presence of stromal and immune cells. This system can maintain primary tumor cells and an associated tumor microenvironment for up to forty-four (44) days following isolation. Importantly, these cultures maintain expression of the soluble tumor markers CA19-9 and CEA similar to those of the primary tumor in patients. Application of this system enabled immune profiling of blood and tumor tissue that found patients with reduced numbers of NK cells and increased presence of immune-related myeloid-derived suppressor cells (MDSC) in their tumors, indicating a potential immunosuppressive profile. Interestingly, we observe reduced levels of CD3 expressing cells in our slice culture despite retention of CD45+ cells. This primary culture model system may be useful for identifying *in vitro* drug response as well as biomarker assessment to help guide personalized therapy and molecular profiles to define predicative molecular profiles of drug responsive tumors.

## RESULTS

### Establishment of tumoroid air-liquid interface (ALI) 3D cultures and tumoroids

We established 3D tumoroid cultures from resected tumor tissues of patients who provided their informed consent and underwent surgery at Fox Chase Cancer Center (Table 1). There were four (4) tissue specimens of colorectal cancer isolated from various segments; two (2) specimens were obtained from the colon and two (2) specimens were obtained from the rectum. We were able to establish both 3D ALI cultures and epithelial tumoroid cultures from three (3) out of four (4) of the tissues that were provided (Table 1). In one case (ReCa030917), we were also able to establish organoid cultures from adjacent normal mucosa. Upon troubleshooting the case (ReCa091516) from which we failed to prepare tumoroid and ALI cultures it was discovered that the specimen that was processed only contained fibrous stroma and was completely devoid of tumor epithelium. A similar situation was also present in the case of the pancreatic cancer specimen (Table 1) where we obtained <0.5 cm^3^ of tumor tissue. Upon histological inspection of H/E stained tissues, approximately 10 - 15% of the tissue-of-origin used for cell culturing contained tumor epithelium with the rest of the tissue specimen containing tumor stroma - a common feature of pancreatic cancers. Although, this did not prevent us from establishing 3D ALI cultures it had a negative effect on establishing epithelial tumoroid cultures that could be subjected to sub-passaging. Tumor tissues were also obtained from three (3) non-small cell lung cancer (NSCLC) patients who underwent surgical resection. These specimens included two (2) squamous NSCLC tissues and one (1) adenocarcinoma NSCLC tissue. We successfully established 3D epithelial tumoroids and ALI cultures from all the lung cancer specimens. However, the squamous NSCLC tumoroids (isolated from two different patients) propagated poorly under our *in vitro* culture conditions and underwent senescence after two (2) passages. By contrast, we were able to propagate tumoroids established from the lung adenocarcinoma (NSCLC) case for eight (8) passages. These lung adenocarcinomas tumoroid cultures were also subjected to drug screening experiments.

**Table 1:** Tumor-of-origin for establishing 3D tumoroid ALI cultures

ID	Anatomical site	Pathology evaluation	Remarks
CoCa091516	Rectum	Rectal cancer	Specimen did not yield growing organoid culture, histopathological evaluation of the provided tissue specimen showed only connective tissue.
CoCa100316	Colon	Colon adenocarcinoma	Organoids and slices were established for short-term culture
CoCa122316	Colon	Colon adenocarcioma	Organoids and slices were established for short-term culture
LuCa121516	Lung	Squamous cell carcinoma (SCC), grade II	Organoids and slices were established for short-term culture. Slow growing organoids.
LuCa122716	Lung, right upper lobe, lobectomy	Adenocarcinoma, Invasive moderately differentiated lung adenocarcinoma, WHO mixed subtype, papillary 70%, micropapillary 20%, and acinar 10%.	Organoids and slices were established for short-term culture. ALI culture show epithelial growth. Epithelial organoids has been subpassaged.
LuCa010517	Lung, left upper lobe, lobectomy	Metastatic Squamous Cell Carcinoma (SCC), grade II	Organoids and slices were established for short-term culture. Slow growing organoids.
PaCa022417	Pancreas	Adenocarcinoma	Low tumor yield, specimen contained mostly connective tissue.
ReCa030917	Rectum	Rectal adenocarcinoma	Tumor tissue content sufficient to establish organoid and 3D cultures
ChCa031017	Liver	Cholangiocarcinoma	Three-dimensional ALI cultures established and 3D early passage organoid culture.

### Growth factor-dependent selection of CRC 3D cultures enrich for tumoroids expressing established tumor markers

**Figure 1:**
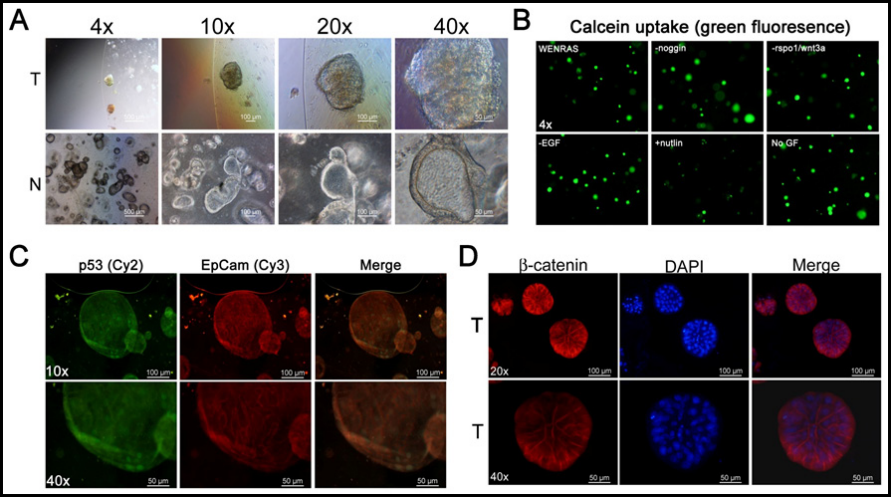
3D colon cancer organoid cultures with growth *in vitro* and expression of tumor-associated markers **A.** Bright field imaging of 3D organoid cultures of normal colon (N) and colorectal cancer (CRC) (T). **B.** Calcein uptake in CRC tumoroid culture subjected to selection using growth factor deprivation. Immunofluorescence for tumor markers p53, EpCam and beta-catenin **C.**, **D.**

The epithelial CRC tumoroid cultures were established from multicellular aggregates in matrigel and grown according to what has previously been described [15] (Figure 1A). In order to promote the selection of cancer cells, WNT3A was excluded from the chemically defined WENRAS media as previously described [3]. One of the CRC tumoroid specimens was relatively insensitive to growth factor deprivation of the WENRAS media including depletion of noggin, EGF, WNT3A and rspo1/WNT3A as determined by calcein uptake (Figure 1B). However, the CRC tumoroids were highly sensitive to treatment with doses of the MDM2 inhibitor nutlin3A to promote selection of CRC tumoroids with mutant Tp53 [16] (Figure 1B). Indeed, this CRC tumoroid expressed the tumor marker EpCam, beta-catenin and nuclear levels of p53 in the absence of p53-triggering stimuli in culture *in vitro* (Figure 1C-1D). Our data reinforce prior published findings that tumoroids expressing tumor-specific markers can be quickly established from small amounts of resected tumor specimens.

### ALI cultures express the tumor markers CA19-9 and CEA

**Figure 2:**
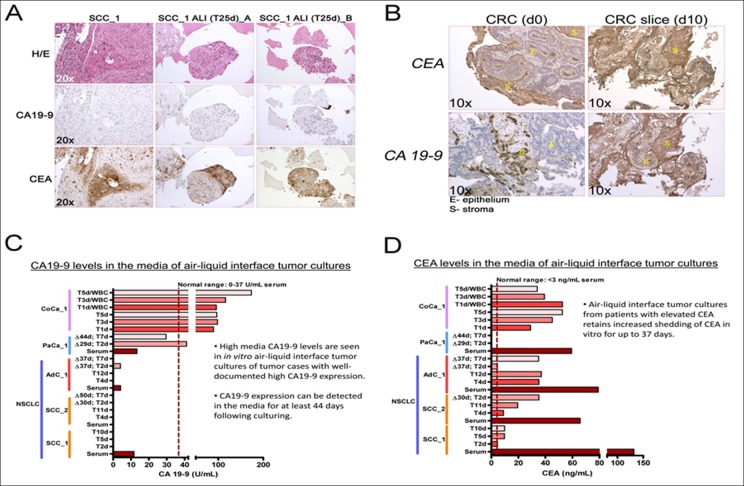
3D air-liquid interface (ALI) lung cancer *in vitro* cultures express several tumor serum markers in culture over extended culture periods **A.**, **B.** IHC for CA19-9 and CEA expression in squamous cell carcinoma and CRC tumor-of-origin and in ALI tumor cultures. **C.**, **D.** Assessment of CEA and CA19-9 in patient serum and 3D culture media over time. Squamous cell carcinoma (SCC) ALI. T1-44d - days in culture following isolation. Δ29d - 29 days before sampling.

To further investigate the expression of tumor markers in 3D tumoroid cultures we chose to analyze the expression of carbohydrate antigen 19-9 (CA19-9) and the carcinoembryonic antigen (CEA) tumor shed antigens (TSAs) commonly used clinically as cancer biomarkers [17–19]. In order to verify tumor-specific expression of these biomarkers, we performed immunohistochemical (IHC) staining of the tumor-of-origin and respective ALI 3D tumor culture (Figure 2A and 2B). Although, the NSCLC culture stained positive for CEA (both squamous cell and adenocarcinoma) they were devoid of staining for CA19-9 (Figure 2A). By contrast, the CRC ALI cultures expressed high levels of both CEA and CA19-9 (Figure 2B). We investigated whether these cultures also shed the biomarkers in the media after prolonged culture and how the levels compare to the levels present in the serum of the patient from which these tumor tissues originated. In order to address this question, we determined the levels of CEA and CA19-9 using ELISA (Figure 2C and 2D). Indeed, as predicted by IHC, 3D ALI tumor cultures with high expression of CA19-9 and/or CEA also shed readily detectable amounts of these biomarkers in their media up to forty-four (44) days after establishing the ALI *in vitro* cultures. From this followed that only cultures from the pancreatic tumor and CRC expressed high levels of CA19-9 in the media whereas 3D ALI cultures of NSCLC did not shed CA19-9 in the media nor was this biomarker abnormally elevated in the serum of such patients (Figure 2C and 2D). Collectively, our data suggest that tumor 3D ALI cultures can retain the expression pattern of clinically relevant tumor biomarkers that are present within the tumor-of-origin for an extended period of time in culture.

### Immune cell profiling of tumors and patient blood reveal differential infiltration of immune cells in donor tissues

**Figure 3:**
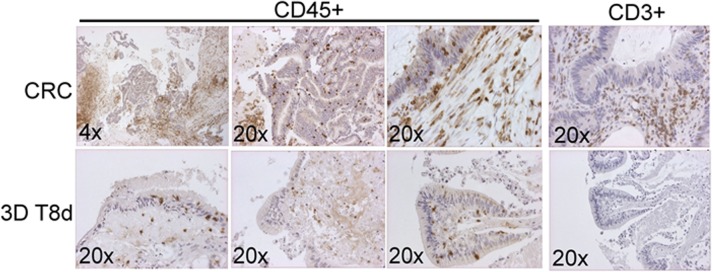
Immune profiling of patient donor tissues and the expression of immune cell markers from 3D ALI cultures *in vitro* IHC for CD45+ and CD3+ cells in a CRC and the corresponding 3D organoid at eight (8) days after initial culture.

A critically important component of the tumor microenvironment is the presence of immune cells that can promote or inhibit tumor progression. In order to address to what extent our organoid 3D ALI cultures contained immune cells with potential importance for tumor etiology or the tumor response to immune oncology drugs we performed immune profiling on blood and tumor tissue from patients from which we established 3D ALI *in vitro* cultures (Figure 3). Data from one patient with lung adenocarcinomas indicated an increased number of myeloid-derived suppressor cells (MDSCs; CD206+/CD33+/HLA-DR-) and double negative (CD4-/CD8-) T cells infiltrating this tumor when compared to the corresponding numbers of these cells in the blood (Table 2). By contrast, the number of tumor infiltrating NK cells and monocytes were significantly reduced within the tumor as compared to the numbers in blood. Taken together this may indicate immune suppression of the tumor-of-origin suggesting that correlating the immune profiles of patients with the donating tumor tissue could yield important information with respect to subsequent analysis of the tumor tissues in the 3D ALI cultures. To address potential differences in longevity of immune cells in our 3D ALI cultures we performed IHC on the cultured tissues (Figure 3). Interestingly, in our CRC *in vitro* cultures we observed the frequent presence of CD45+ cells in the tumor-of-origin. Indeed, CD45+ cells were also present in the cultured tissue following eight (8) days of *in vitro* 3D culture (Figure 3). By contrast, we observed a marked drop in the number of CD3+ cells as compared to the tumor-of-origin following the same length of culturing. This would suggest that although CD45+ hematopoietic cells are present in both the tumor-of-origin and the 3D cultures of CRC tissue, there is a marked reduction in the number of CD3+ cells under these culture conditions.

**Table 2:** Immune cells in blood and tumor specimen used to establish 3D cultures

Immune cells	LuCa122716 (NSCLC, adenocarcinomas)	
	Blood (%)	Tumor (%)
**Myeloid cells (CD45+/CD206+)a**		
MDSC (CD33+/HLA-DR-)	0.41^a^	1.20^a^
Monocytes, CD16+ (CD14+/CD16+)	19.7^a^	0.32^a^
Monocytes, CD16- (CD14+/CD16-)	47.7^a^	1.98^a^
**T cell(CD45+/CD3+)b**		
T-cell, CD4+	30.0^b^	29.9^b^
T-cells, CD8+	42.2^b^	50.0^b^
T-cells, CD4-/CD8-	17.7^b^	6.83^b^
T-cells, CD4+/CD8+	10.0^b^	13.2^b^
**NK cell (CD45+/CD56+)c**		
Immature (CD117+/CD94-)	0.075^c^	2.07^c^
CD16+/CD328+	65.9 ^c^	19.7 ^c^

^a^Percent of Myeloid cells (CD45+/CD206+)

^b^Percent of T cell population (CD45+/CD3+)

^c^Percent of NK cells (CD45+/CD56+)

### Evaluation of cytotoxic responses in cancer 3D organoid cultures reveals differential responses to FDA-approved drugs

**Figure 4:**
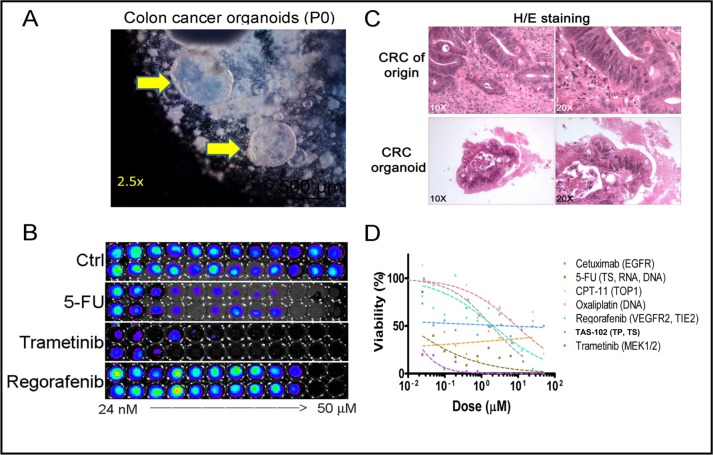
Assessment of CRC patient tumoroid responses to cytotoxic drugs *in vitro* at 2 weeks following tumor resection . **A.** Bright field imaging of 3D organoid cultures from a colorectal cancer (CRC). **B.** CellTiter-Glo (ATP content) was used to determine the dose-response characteristics of a CRC tumoroid following treatment with FDA-approved drugs and trametinib. A duplicate 12-point dose titer was used for each drug that was investigated with 5-10 organoids per 96-well. **C.** H/E staining of the CRC of origin and tumor organoid cultures. **D.** Dose-response relationship of FDA- approved drugs and trametinib in CRC tumoroid cultures.

To investigate drug responses in the 3D cultures, we established 3D tumoroids from a resected colon adenocarcinoma (Figure 4A) and cultured these in the absence of WNT3A as previously shown (Figure 1B). These cultures retained some of the cytological hallmarks of the tumor-of-origin as evident by H/E staining (Figure 4C). Next, we subjected these tumoroid cultures to screening using FDA-approved compounds commonly used to treat colorectal cancer. We also included the MEK-inhibitor trametinib which is not yet approved for CRC therapy but has shown promising single-agent activity in a previously published drug screen of CRC tumoroids [3] (Figure 4B). After five (5) days of culture we found that CRC patient tumoroids are particularly sensitive to treatment with the approved drugs 5-fluoruracil (5-FU) and trametinib. Furthermore, regorafenib, cetuximab and irinotecan (CPT-11) in descending order provided similar dose-response profiles but with higher IC_50_ values as compared to 5-FU. Oxaliplatin and TAS-102 (trifluridine/tipiracil) did not yield interpretable dose-response profiles for the drug concentrations that were tested. Our data indicates that 3D tumoroid *in vitro* cultures can be interrogated in a high-throughput format using cytotoxic drugs within weeks following surgery.

## DISCUSSION

Recent advances in 3D tumoroid culture methodology have enabled scientists to replicate the complex tumor organ system composed of multiple cell lineages including tumor epithelium, stromal cells and vasculature *in vitro* to better study the complex interaction of these cells in supporting tumor growth and potential drug response. Both the normal and tumor epithelium can now be readily cultured from biopsies, propagated and expanded *in vitro* using defined culture conditions. Advances in generating tumoroids from pancreatic cancer [20], glioblastoma [21], prostate cancer [22, 23] and colorectal cancer [3] reveal that patient-derived tumoroids recapitulate patient-specific histological features. These tumoroids can also be cryopreserved in a similar manner as continuous cell lines and can subsequently be characterized by genomics, transcriptomics and using high-throughput *in vitro* drug screening assays, something that has spurred initiatives to develop live patient-derived tumoroid biobanks [1]. It is possible that data from tumoroid drug screening assays not only will complement and corroborate tumor genomic data but may in addition provide prediction on drug responses where sequencing data alone might fall short. Unfortunately, at this time the tumoroid systems are limited in their capacity to predict responses to novel promising immune oncology drugs since they promote the expansion of the tumor epithelium and do not support the maintenance and expansion of patient-specific immune and stromal cells that contribute to an immune suppressive tumor microenvironment.

Here we show that 3D ALI tumoroid cultures that include a patient-specific stromal component can be maintained from resected solid tumor specimens when cultured in a Matrigel matrix for up to forty-four (44) days as determined by the expression of TSA biomarkers CEA and CA19-9 in the media. Previous studies on mouse neonatal small intestinal specimens indicate that tissue can be preserved for at least 350 days when isolated from young mice using a similar system [13]. The longevity estimate here would suggest that the living tumor cells are being preserved in our system and it may be possible to extend the life of the cell population. Thus, our finding might be less surprising considering the previous discoveries. However, it remains to be addressed to what extent the different cell populations are maintained in the stroma when compared to the donating tumor-of-origin. For example, although we readily detect CD45+ cells in the 3D ALI cultures, a clear reduction in the presence of CD3+ cells in the tissues was observed following eight (8) days in culture. Moreover, we were only able to document outgrowth of tumor-associated fibroblasts in one of our squamous 3D ALI NSCLC cultures suggesting that the culture conditions did not support the long-term expansion of the stromal compartment. The above might be less surprising since the chemically-defined WENRAS media employed in this study do not contain growth factors that can support for myeloid and lymphocytic cells, including FGF and interleukins. In fact, one component of the WENRAS media is the signaling peptide noggin which acts as an inhibitor of the bone morphogenic protein-4 (BMP-4), a member of the transforming growth factor-beta (TGF-beta) superfamily [24]. BMP signaling limits hyper-proliferation of the intestinal epithelium and maintains differentiation along the crypt-villus axis by forming a gradient of BMP-activity where mesenchymal cells beneath the crypts express high levels of noggin whereas high levels of BMP-2 and-4 are expressed by mesenchymal cells both in the crypt and the villi [25–28]. Recent evidence has also shown that BMP-signaling constrains the self-renewal of Lgr5+ cells via SMAD-mediated repression of the stem cell signature [29]. SMAD4 expression is frequently lost in invasive colorectal cancer, indicating that the quenching of the BMP-signaling pathway in CRC tumoroids through noggin supplementation may be less essential for successful tumoroid *in vitro* culture [30, 31]. Indeed, one of our CRC tumoroid cultures was completely refractory to the withdrawal of recombinant noggin from the WENRAS media. Thus, further optimization of the growth factors that constitute the WENRAS media is likely necessary to support the diverse cell types that constitute the tumor stroma.

In conclusion, tumor 3D ALI cultures retain the capacity to maintain tumor stroma and characteristics of the primary tumor including the long-term production of CEA and CA19-9 TSAs (>44 days) following isolation. CA19-9 and CEA are commonly used to monitor CRC in the clinic. In our system, the pancreatic cancer specimens expressed high levels of CA19-9 consistent with this being a biomarker often used to detect and monitor treatment of patients with pancreatic cancer. Profiling of immune cell content in the blood and tumors from patients have identified varying populations of myeloid-derived suppressor cells (MDSC), double-negative T cells (DNT), mature NK cells, and monocytes across each tumoroid that appear to correlate with growth and drug response. The fact that these cultures maintain the production of TSAs makes tumoroids useful to better understand the effects TSAs have on tumor immune suppression. Recently, Kline *et al*. have shown that the CA125 TSA is immunosuppressive to antibody-based tumor therapies [32]. This system will enable better understanding of how the alternative immunosuppressive mechanism can affect the growth and stasis of tumors in the presence of of a given patient's immune system. Additional studies are being designed to better understand the relationship of these cell subtypes and response to therapeutics with the immune microenvironment within the tumoroid cell culture.

## MATERIALS AND METHODS

### Tissue isolation and blood collection

Surgically resected tumor tissues were obtained from eleven (11) patients subjected to treatment at Fox Chase Cancer Center (FCCC). Patient material was collected from a total of five (5) colorectal cancer cases, three (3) NSCLC cancer cases, two (2) cases of pancreatic cancer and one (1) case of cholangiocarcinoma. Tissue fragments representative of what was used for *in vitro* culture were sampled and fixed in formalin overnight. The remaining tissues were chopped into approximately 5-mm pieces and washed with cold PBS as previously described [15, 33]. Blood was collected in K2-EDTA blood collection tubes (BD Vacutainer Lavender) and serum blood collection tube (BD Vacutainer Red Top) from each patient immediately prior to surgery. Blood was subjected to RBC lysis solution (BioLegend) to remove red blood cells and washed in ice cold PBS/2%FCS. The remaining WBCs were stored at 4°C in Streck Cell Preservative™ (Thermo Fisher Scientific) and subsequently analyzed by flow cytometry. Blood was also allowed to clot and the serum was separated by centrifugation for 10 minutes at 3,500 rpm, aliquoted and stored at −80°C. All experiments with patient tissues were performed according an approved protocol from the Institutional Review Board of FCCC and all patient tissue or blood samples were obtained with informed consent.

### Establishment of 3D air-liquid interface (ALI) & tumoroid cultures

ALI cultures were generated in a similar manner as previously described [13]. Cell culture inserts (Millicell-CM, Millipore) for 24-well plates were coated with Matrigel (BD bioscience) to generate an acellular bottom layer. The Matrigel was allowed to solidify for 10 minutes at 37°C. The tumor tissue fragments were washed in ice-cold PBS and minced rapidly by using iris scissors on a cold tissue culture plate to prevent drying of the tissue. Approximately 30-50 mg of minced tissue was mixed with 400 μL of ice-cold Matrigel by pipetting using a 2.0 ml pipette and transfer to the cell culture insert. The tissue was allowed to solidify for 10 minutes at 37°C. The outer dish was subsequently filled to cover the acellular layer with WENRAS media [14, 30] (advanced Dulbecco's modified Eagle medium/F12 [Invitrogen] supplemented with penicillin/streptomycin [Invitrogen], 10 mmol/L HEPES [Invitrogen], Glutamax [Invitrogen], 1 x B27 [Invitrogen], 1 mmol/L N-acetylcysteine [Sigma] containing the following growth factors: gastrin, nicotinamide, EGF, recombinant noggin, WNT3A- and RSPO1-conditioned media. The media was also supplemented with A83-01, SB202190 and Y-27632 as described previously [15]. Tumoroid cultures were prepared by washing the tissue fragments 6-8 times in 10 mL of ice-cold PBS allowing the tissue to settle after each wash and removing the supernatant. The fragments were incubated in digestion buffer containing collagenase IV and dispase II for approximately one (1) hour at 37°C. The supernatant was collected in a 15-ml tube and centrifuged for 3 minutes at 200 x g. The supernatant was collected and tumor infiltrating immune cells were enriched by using Percoll separation as previously as described [34] for immune cell profiling (see below). The supernatant was washed in PBS once, the pellet was dissolved and the number of tissue fragments were counted, seeded in 40 μl of Matrigel at a density of 25-40 tumoroids/24-well and cultured in WENRAS medium. The WENRAS media was changed every other day and the tumoroids were passaged at a 1:2 - 1:6 ratio approximately once every week as previously described [15] using TrypLExpress (Invitrogen) supplemented with Y-27632 (Sigma-Aldrich).

Drugs used in this study were obtained from the following sources: Cetuximab (Fox Chase Cancer Center Pharmacy), 5-FU (Fox Chase Cancer Center Pharmacy), CPT-11 (Fox Chase Cancer Center Pharmacy), oxaliplatin (Fox Chase Cancer Center Pharmacy), regorafenib (MedKoo, catalogue #202436), TAS-102 (MedKoo, catalogue #205641), trametinib (MedKoo, catalogue #201458).

### ELISA

Serum samples and media were collected at various time points following 3D tumoroid culture. The total protein content was determined in each sample using the BSA Protein Assay Kit (ThermoFisher Scientific). The protein levels were normalized and subsequently subjected to ELISA for human CEA (ThermoFisher Scientific) and human CA19-9 (ThermoFisher Scientific). The protein standards, media and serum samples were diluted according to the manufacturer's instructions. The resulting colorimetric reaction was analyzed at 562 nm using a Beckman Coulter DTX880 Multimode Detector. The levels of protein present in each sample were determined mathematically from the straight-line equation of the standard curve.

### Histopathology and immunohistochemistry

Tumoroids and 3D ALI tumor cultures were fixed in formalin and embedded in paraffin. Cut sections were stained with H/E. Slide sections were rehydrated and subjected to antigen retrieval in citric acid buffer (1.0 mM, pH 6.0). Endogenous peroxidases were blocked with 3% hydrogen peroxide. The slides were blocked for 30 minutes in 5% goat serum and subsequently incubated with the primary rabbit antibodies towards CD45 (ab10559, AbCam), CD3 (ThermoFisher Scientific), CEA (A0115, Dako) and CA19-9 (M3517, Dako) at room temperature overnight. Slides were then washed and incubated with secondary peroxidase conjugated antibodies using the ImmPRESS™ HRP Anti-Rabbit/Mouse IgG (Peroxidase) Polymer Detection Kit (Vector Laboratories). Following washes in PBS, the slides were stained using the 3,3’-diaminobenzidine tetrahydrochloride (DAB) Horseradish Peroxidase (HRP) Substrate Kit, 3,3’-diaminobenzidine (Vector Laboratories). The slides were counterstained using hematoxylin.

### Profiling of immune cells in patient blood and tumor tissue

White blood cells (WBC) and tumor-infiltrating cells were divided into a myeloid staining category (CD80, CD68, CD206, CD16, HLA-DR, CD14, CD33, CD45 and propidium iodide [PI]) and a T-cell staining category (CD329, CD328, CD94, CD16, CD4, CD117, CD56, CD3, CD8, CD45 and PI). Stained cells were analyzed on a Beckton-Dickinson (BD) ARIA II flow cytometer with four lasers at 633-, 488-, 405- and 365-nm wavelengths (Cell Sorting Facility, Fox Chase Cancer Center). Data were collected with BD FACS Diva software version 6 and analyzed with Flowjo v9.2 (Tree Star Inc.). Single-cell events were first gated by a forward scatter height versus forward scatter area plot and viable CD45+ cells were then gated by lack of PI staining and analyzed for the surface markers indicated for the myeloid and NK/T-cell category.

## Abbreviations

ALI: air-liquid interface
BMP: bone morphogenic protein
CA19-9: carbohydrate antigen 19-9
2D: two-dimensional
3D: three-dimensional
CEA: carcinoembryonic antigen
CA19-9: carbohydrate antigen 19-9
CRC: colorectal cancer
DAB: 3,3’-diaminobenzidine tetrahydrochloride
H/E: hematoxylin-eosin
HRP: horseradish peroxidase
NSCLC: non-small cell lung cancer
PI: propidium iodide
WBC: white blood cells
TSA: tumor shed antigen
